# Smear positive pulmonary tuberculosis and its risk factors among tuberculosis suspect in South East Ethiopia; a hospital based cross-sectional study

**DOI:** 10.1186/1756-0500-7-285

**Published:** 2014-05-06

**Authors:** Begna Tulu, Nagasa Dida, Yibeltal Kassa, Biruhalem Taye

**Affiliations:** 1 Microbiology, Immunology and Parasitology Department, College of Medicine and Health Sciences, Bahir Dar University, P. O. Box 79, Bahir Dar, Ethiopia; 2 Microbiology, Infectious Diseases and Parasitology Department, College of Medicine and Health Sciences, Madawalabu University, P. O. Box 302, Bale-Goba, Ethiopia; 3 Department of Public Health, College of Medicine and Health Sciences, Madawalabu University, P. O. Box 302, Bale-Goba, Ethiopia; 4 Department of Nursing, College of Medicine and Health Sciences, Madawalabu University, P. O. Box 302, Bale-Goba, Ethiopia; 5 Tropical and Infectious Diseases Department, Aklilu Lemma Institute of Pathobiology, Addis Ababa University, P. O. Box 1176, Addis Ababa, Ethiopia

**Keywords:** Smear positive PTB, Prevalence, Risk factors

## Abstract

**Background:**

Tuberculosis remains a deadly infectious disease, affecting millions of people worldwide. Ethiopia ranks seventh among the twenty two high tuberculosis burden countries. The aim of this study was to determine the prevalence of smear positive pulmonary tuberculosis and its associated risk factors in Goba and Robe hospitals of Bale zone.

**Methods:**

A cross-sectional study was conducted on tuberculosis suspected patients from February-May 2012. Sputum samples were examined for acid fast bacilli using Ziehl-Neelsen staining and interview was conducted for each patient. Descriptive statistics, binary logistic and multivariable logistic regression analyses were employed to identify factors associated with pulmonary tuberculosis infection.

**Result:**

The prevalence of smear positive tuberculosis was 9.2%. Age >36 (AOR = 3.54, 95% CI = 1. 3–9.82), marital status (AOR = 8.40, 95% CI = 3.02-23.20), family size (AOR = 4. 10, 95% CI = 1.60-10.80), contact with active tuberculosis patient (AOR = 5. 90; 95% CI = 2. 30–15.30), smoking cigarette regularly (AOR = 3. 90; 95% CI = 1. 20–12.40), and human immunodeficiency virus sero-status (AOR = 11. 70; 95% CI = 4. 30–31.70) were significantly associated with smear positive pulmonary tuberculosis.

**Conclusion:**

The prevalence of smear positive pulmonary tuberculosis was high in the study area. Age, marital status, family size, history of contact with active tuberculosis patient, smoking cigarettes, and HIV sero-status were among the risk factors significantly associated with acquiring tuberculosis. Hence, strict pulmonary tuberculosis screening of HIV patients and intensification of health education to avoid risk factors identified are recommended.

## Background

Tuberculosis (TB) is still one of the infectious diseases known by its significant cause of morbidity and mortality affecting millions of people worldwide. Although it is a global epidemic, TB predominantly affects developing countries, where 98% of worldwide TB death occurs [1,2]. Resource poor countries share the global burden with highest rates, with an estimated 55% of global case in Asia and 31% in Africa [2-5]. According to the WHO Global TB Report, Ethiopia ranks seventh among the world’s 22 high-burden TB countries. The estimated incidence rate was 261 cases per 100,000 population and 29 thousand deaths in 2010, with an estimated prevalence rate of 394 cases per 100,000 populations [2].

Studies have shown that human immunodeficiency virus (HIV)/AIDS, diabetes, cancer, malnutrition, alcoholism, smoking cigarette, active TB contact, extreme poverty, homelessness, and being in prison were among the commonly identified risk factors associated with tuberculosis in most developing countries including Ethiopia [6-9].

In Ethiopia, a study conducted in Metehara sugar factory hospital showed 14.2% prevalence of smear positive pulmonary tuberculosis [10]. Another study on smear positive TB conducted in Agaro health centre showed a prevalence of 10.9% [11]. However, the rate of pulmonary TB (PTB) and its associated risk factors in the study area were not known. Therefore, the purpose of this study was to determine the prevalence of smear positive PTB and its associated risk factors among PTB suspected patients attending Bale Goba and Robe Hospitals.

## Methods

A cross-sectional study was conducted in Bale Goba and Robe Hospitals of Bale zone, which are 444 km and 432 km away from the capital city, Addis Ababa, respectively towards the South East part of Ethiopia. The study was conducted from February 01 to May 25, 2012. Sample size was calculated using single population proportion formula [n = (Z α/_2_) ^2^ P (1-P) /d^2^], where, n = sample size, Zα/_2_ = statistic for the level of confidence at 95%, which is 1.96, P = expected prevalence which was set 50% to yield maximum sample size, and d = precision which is 5%. Including 10% non response rate the sample size became 420. However, 391 of them participated with none response rate of 93.1.”

Pretested questionnaire was used to collect information on socio-demographic characteristics and other associated risk factors. Data collectors were also trained for one day on the instrument of data collection and how to collect the sputum specimen and also supervised by the investigators during the entire data collection.

Three consecutive sputum samples, i.e. spot-morning-spot were collected from each of the PTB suspected cases. Experienced laboratory technologists performed the laboratory test following the standard procedure of Ziehl-Neelsen staining technique recommended by WHO [12,13]. The completeness of the questionnaire was checked and the examination sputum samples were performed both in the hospital's laboratory and Madawalabu University Biomedical Laboratory for quality control. Provider initiative counseling and testing (PIHCT) for HIV is being practiced for all TB suspects hence, HIV results were collected from hospital records with patient card numbers.

The data from questionnaires and laboratory results were analyzed using statistical software (SPSS package version 16). The results were summarized using descriptive statistics including frequencies and mean. Difference of proportion was evaluated using Chi-square test and p < 0.05 was considered as significant. A binary and multiple logistic regression analysis were used and odds ratio was calculated to determine the strength of association between variables and lifetime exposure to TB infection.

The study was approved by Research Ethics and Review Committee of Madawalabu University. In addition, official permission was obtained from the administration offices of Goba and Robe Hospitals. Furthermore, the objective of the study was explained to the participants and written consent was obtained before specimen collection. All participants who were affected by the disease were treated in the hospital accordingly.

## Result

### Socio-demographic characteristics of tuberculosis suspected patients

Out of the total study participants, 55.1% were residence of urban setup, and 52.9% were males. The mean age of the study participants was 40.8 years (std. deviation ± 20. 2). Concerning their marital status, 71.9% were married. Illiterate study participants constitute 50.1%, followed by primary education (1–8) which was 33.8%.

Regarding their living situation, 98.2% of the study participants live together with their family or relatives while the rest 1.8% of them live in prison and on the street. A majority, 57.5% of the study participants reported that they had family member of five or less while the rest 42.5% of them posses a family member of more than five. With regard to the number of rooms in the respondents’ home, 89.0% of them reported they have three or less rooms while the rest reported they have more than three. The predominant occupational status of the study participants was unemployed 43.4%, followed by farmer 38.1% (Table 1).

**Table 1:** Socio-demographic characteristics and the proportion of smear positive tuberculosis in different categories of the study subjects in Goba and Robe Hospitals, 2012

**Characteristics**		N (%) SPTB^*^	P value^**^
**Residence**	Urban	26(72.2)	0.029
	Rural	10(27.8)	
**Sex**	Male	20(55.6)	0.742
	Female	16(44.4)	
**Age (years)**	≤15	0	0.005
	16-35	8(22.2)	
	≥36	28(77.8)	
**Marital status**	Unmarried***	16(44.4)	0.022
	Married	20(55.6)	
**Educational status**	Illiterate	16(44.4)	0.331
	Grade 1-8	16(44.4)	
	Grade 9 and above	4(11.1)	
**Living Situation**	Living in prison & on the street	3(8.3)	0.002
	Living with family	33(91.7)	
**Family size**	>5	22(61.1)	0.017
	≤5	14(38.9)	
**Number of rooms**	≤3	34(94.4)	0.273
	>4	2(5.6)	
**Occupation**	Employee	6(16.7)	0.290
	Unemployed	12(33.3)	
	Farmer	18(50.0)	

*Smear positive PTB, ** χ^2^ test for trends *** Single, widowed and divorced.

### Prevalence of smear positive pulmonary tuberculosis

The prevalence of smear positive PTB supported by chest x-ray in the study population was 9.2%. The result also showed 39.0% of the study participants were co-infected with smear positive PTB and HIV. Urban residents were more frequently infected with smear positive PTB than compared to rural ones (p = 0. 029). A significantly higher proportion of smear positive TB was reported among older ages, ≥ 36 years (p = 0. 005). Smear positive PTB was also more frequently reported among those having > 5 family member (p = 0. 017). Similarly, unmarried individuals were found to be more frequently infected with smear positive PTB compared to married ones (p = 0. 022) (Table 1).

### Risk factors associated with smear positive pulmonary tuberculosis

Based on bivariate logistic regression, HIV sero-status, smoking cigarette regularly, and history of contact with the active TB patient were significantly associated with smear positive PTB (OR = 10.50, 95% CI = 4.80-22.80, OR = 4.20, 95% CI = 1.80-9.80, and OR = 2.40, 95% CI = 1. 20–5.10 respectively). However, previous TB infection, regular alcohol use, repeated respiratory tract infections, family history of TB, and diabetic cases were found to be statistically insignificant (p > 0.05) with smear positive PTB (Table 2).

**Table 2:** Exposure status to different TB risk factors and proportions of TB positive participants at Goba and Robe Hospitals, 2012

**Characteristics**	**N (%) SPTB**	**Crude OR (95% CI)**	**P-value**
Previous TB infection			
Yes	8(22.2)	1.30[0.56-2.90]	0.544
No	28(77.8)	1	
Family History of TB			
Yes	10(27.8)	2.10[0.96-4.60]	0.064
No	26(72.2)	1	
Contact with active TB patient			
Yes	12(33.3)	2.40[1.20-5.10]	0.021
No	24(66.7)	1	
Smoking cigarette regularly			
Yes	9(25.0)	4.20[1.8-9.80]	0.001
No	27(75.0)	1	
Drinking alcohol regularly			
Yes	5(13.9)	1.00[0.38-2.74]	0.974
No	31(86.1)	1	
HIV sero-status			
Positive	16(44.4)	10.50[4.80-22.80]	<0.001
Negative	20(55.6)	1	

After adjusting for those significantly associated variables, age (p = 0. 015), marital status (p < 0.001), family size > 5 (p = 0. 004), history of active TB patient contact (p < 0.001), history of smoking cigarette (p = 0. 021) and HIV sero-status (p < 0.001) remains significant associated with smear positive PTB. However, factors like place of residence, and living situations were not significantly associated with smear positive PTB with (p > 0.05) (Table 3).

**Table 3:** Adjusted Odds Ratio of exposure status to different TB risk factors and proportions of TB positive participants at Goba and Robe Hospitals, 2012

**Characteristics**	**N (%) SPTB**	**Crude OR (95% CI)**	**P-value**	**Adjusted OR (95% CI)**	**P- value**
Residence					
Urban	26(72.2)	2.28[1.07-4.87]	0.033	-	-
Rural	10(27.8)	1		-	
Age (yrs)					
16-35	8(22.2)	1		1	
>36	28(77.8)	2.97[1.32-6.71]	0.032	3.54[1.3-9.82]	0.015
Marital status					
Unmarried	16(44.4)	2.22[1.11-4.47]	0.025	8.40[3.02-23.20]	<0.001
Married	20(55.6)	1		1	
Living Situation					
In prison & on street	3(8.3)	7.98[1.71-37.20]	0.008	-	-
With family	33(91.7)	1		-	
Family size					
>5	14(38.9)	2.30[1.10-4.70]	0.020	4.10[1.60-10.80]	0.004
≤5	22(61.1)	1		1	
Active TB patient contact					
Yes	12(33.3)	2.40[1.20-5.10]	0.021	5.90[2.30-15.30]	<0.001
No	24(66.7)	1		1	
Smoking cigarette					
Yes	9(25.0)	4.20[1.8-9.80]	0.001	3.90[1.20-12.40]	0.021
No	27(75.0)	1		1	
HIV sero-status					
Positive	16(44.4)	10.50[4.80-22.80]	<0.001	11.70[4.30-31.70]	<0.001
Negative	20(55.6)	1		1	

Respondents who were above the age of 36 were about four times (AOR = 3. 54, 95% CI = 1. 3–9.82) more likely to develop smear positive PTB compared to those younger ages. Similarly, unmarried respondents were about eight times (AOR = 8. 40, 95% CI = 3.02-23.20) more likely to develop smear positive PTB than married ones. Those who had contact with active TB patient in their vicinity were about six times (AOR = 5. 90; 95% CI = 2. 30–15.30) more likely to develop smear positive TB than those who were not. Those who reported smoking cigarette regularly were also about four times (AOR = 3. 90; 95% CI = 1. 20–12.40) more likely to develop smear positive TB than those who do not smoke. Concerning HIV sero-status, those who were HIV positive were about twelve times (AOR = 11. 70; 95% CI = 4. 30–31.70) more likely to develop than who were HIV negative (Table 3).

## Discussion

This study tries to provide insights into the prevalence of smear positive PTB among tuberculosis suspected patients attending Bale Robe and Goba hospitals, as well as outline some possible risk factors. The results showed 9.2% prevalence of smear positive PTB. This shows that TB is still one of the major public health concerns in the study area and in Ethiopia as well. Similar studies conducted in Rwanda reported (17.3%) [5], and in Nigeria (14.7%) [14] which was significantly higher compared to the findings in this study. This may be due to the community based program launched several years ago using home to home community health workers, who are capable of creating awareness better than the previous strategies in Ethiopia. The result in this study was in line with similar study conducted in hospitals and health centers of Ethiopia; Agaro Teaching Health Center, (10.9%) [11], Jimma University Specialized Hospital, (8.5%) [15], and in Jimma Seka (10.6%) [16]. However, the finding of the present study showed lower prevalence than similar study conducted in Tigray (17.7%) [17], in Addis Ababa (21.3%) [18], and in Eastern Ethiopia 14.2% [10]. This could be associated to differences in awareness level of the patients leading to passive detection in those who had sign and symptoms of the disease.

This study also indicated that the prevalence of smear positive PTB was significantly higher in HIV positive patients than HIV-negative. Out of the total 36 smear positive pulmonary tuberculosis 44.4% were HIV positive. Similarly, a number of studies conducted in different parts of the world and some other African countries showed the prevalence rate of co-infection ranges from 8% in Congo to 82% in Swaziland [3,5,19]. In Ethiopia, based on the WHO report in 2012 the rate of co-infection was (41%) [2]. As it is well established the strong association between HIV and TB is attributed to the overlapping of the age group that both infections are affecting and the immunological deprivation that HIV results in.

As far as sex is concerned, similar studies conducted in Ethiopia and other countries like Rwanda, and Myanmar, reported that smear positive PTB is more common among men than women [2,5,15,20-22]. In the present study, the prevalence of smear positive tuberculosis was not statistically significant between sexes, though a relatively higher prevalence was reported among men 55.6%, and women 44.4%. Similar to previous studies conducted in Ethiopia and other countries [5,15,20,21], smear positive PTB affects mostly adults in the economically productive age groups; around two-thirds of cases were estimated to occur among people aged 15–59 years.

Urban dwellers were found to be more associated with smear positive TB in this study. The prevalence of smear positive PTB was 72.2% among urban dwellers compared to 27.8% among the rural dwellers. This finding is consistent with similar studies conducted in India (69.2%) and Ethiopia (52.7%) [9,23]. This might be due to the fact that urban set up is characterized by overcrowding and suffocation which is an important risk for respiratory disease including tuberculosis.

The role of active smoking in the development of TB is well known [2,6], the same is true in this study in which active smoking was significantly associated with smear positive TB with (p = 0. 021). A history of active TB patient contact and HIV- sero status was also among the risk factors that showed significant association with smear positive TB (P < 0.005). This is consistent with WHO report and studies conducted in different parts of the world [2,6,24].

Some of the limitations of this study include direct smear microscope alone may underestimate the prevalence of PTB in the study population. Other techniques like culture and molecular assays could best estimate the prevalence of PTB among the study subjects. However, due to financial constraints, we were unable to perform these advanced tests. Selection bias may also arise from convenience sampling.

## Conclusion

In conclusion, the prevalence of smear positive PTB among tuberculosis suspect was high (9.2%) in Bale Goba and Robe hospitals. Smear positive PTB was more frequently reported among urban residence, age > 36 years, male sex, and unemployed individuals. Risk factors like HIV sero-status, active TB patient contact, and cigarette smoking were significant to acquire smear positive TB. Hence, we recommend strict PTB screening of HIV patients, and health education to avoid smoking. It would be better to expand health education to household contacts of smear positive PTB patients on how to protect them from TB infection. Finally, we would also like to recommend longitudinal studies with more advanced laboratory techniques.

## Competing interest

None of the authors and other organizations has competing interest.

## Author’s contribution

BT* and ND conceptualized and designed the study. BT and YK assisted in designing the study design. BT*, ND and YK analyzed and interpreted the data, drafted the manuscript and critically reviewed the manuscript. BT assisted in drafting and critically reviewing the manuscript. All the authors’ read and approved the manuscript.
